# Design, synthesis, cytotoxic activity, and apoptosis inducing effects of 4- and N-substituted benzoyltaurinamide derivatives

**DOI:** 10.3906/kim-2009-1

**Published:** 2020-12-16

**Authors:** Özlem AKGÜL, Mümin Alper ERDOĞAN, Derviş BİRİM, Çağla KAYABAŞI, Cumhur GÜNDÜZ, Güliz ARMAĞAN

**Affiliations:** 1 Department of Pharmaceutical Chemistry, Faculty of Pharmacy, Ege University, İzmir Turkey; 2 Department of Physiology, School of Medicine, İzmir Katip Çelebi University, İzmir Turkey; 3 Department of Biochemistry, Faculty of Pharmacy, Ege University, İzmir Turkey; 4 Department of Medical Biology, Faculty of Medicine, Ege University, İzmir Turkey

**Keywords:** Apoptosis, Bcl-2 family proteins, caspase-3/9, cytotoxicity, taurine

## Abstract

In this study, a group of 4-substituted benzoyltaurinamide derivatives were designed, synthesized, and investigated for their anticancer activity against three cancer cell lines and one nontumorigenic cell line by MTT assay. Among the final compounds, methoxyphenyl derivatives 14, 15, 16 were found to be effective against all the tested cancerous cell lines with promising selectivity. The most active compounds were further evaluated to determine the molecular mechanism of their anticancer activity by using western blot assay and the Annexin V-FITC/PI test. Compound 14 (in SH-SY5Y and MDA-MB-231 cell lines) and 15 (in SH-SY5Y cell line) were found to induce intrinsic apoptotic pathway by upregulating BAX, caspase-3, and caspase-9, while downregulating Bcl-2 and Bcl-xL expression levels. According to mechanistic studies, compounds displayed their anticancer activity via three different mechanisms: a. caspase-dependent, b. caspase-independent, and c. caspase-dependent pathway that excluded caspase-9 activation. As a result, this study provides interesting data which can be used to design new taurine-based anticancer derivatives.

## 1. Introduction

Since cancer is one of the leading causes of death worldwide, extensive resources have been invested in the development of increasingly potent anticancer agents for clinical use. Despite the advances, issues related to drug toxicity and resistance during treatment persist, thereby promoting researchers to develop more efficient, safer therapeutic agents [1–3].

Cancer, characterized by the abnormal proliferation of genetically altered cells, exhibits certain hallmarks, namely, sustained proliferative signaling, evasion of growth suppressors, replicant immortality, resistance to programmed cell death (apoptosis), induction of angiogenesis, and invasion of healthy tissue [4]. Since apoptosis is the programmed cell death process responsible for the controlled eradication of damaged cells, it plays a crucial role in regulating tissue homeostasis, balancing cell death and survival, and regulating cell proliferation [5]. Generally, receptor-mediated extrinsic and mitochondrial-dependent intrinsic pathways trigger the activation of caspase-3, thereby resulting in apoptosis. In the intrinsic pathway, proapoptotic (i.e., BAX and BAK) and antiapoptotic (i.e., Bcl-2, Bcl-xL, Bcl-w, Mcl-1, and Bfl1/A1) proteins stabilize the outer membrane of the mitochondria and activate caspase-9 and caspase-3 [6]. Studies indicate that cancer cells evade apoptosis by deregulating this process, thereby resulting in increased drug resistance. Given this challenging situation, apoptotic proteins have become the prime target for anticancer research [7,8].

**Figure 1:**
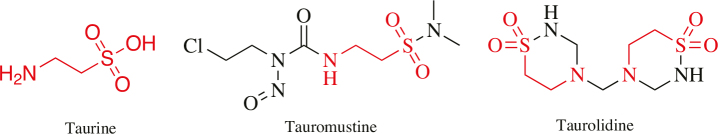
Taurine and its biologically active derivatives.

**Figure 2:**
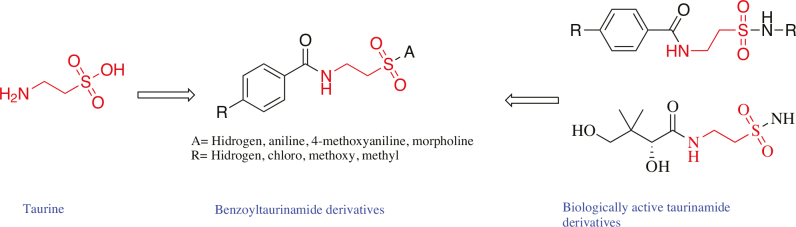
Schematic representation of drug design strategy.

Taurine (i.e. 2-aminoethanesulfonic acid) is the only endogenous amino acid that does not interfere with protein synthesis (Figure 1). It is renowned for its cytoprotective effects and is involved in diverse physiological processes, including membrane stabilization, osmoregulation, neuromodulation, regulation of calcium homeostasis, and antioxidation [9]. Additionally, multiple studies have reported radioprotective, antimutagenic, and chemo-preventive properties associated with this compound [10]. Taurine exhibits anticancer activity via the elevation of caspase-3 and caspase-9 expression levels and BAX/Bcl-2 ratios [11,12]. It also induces apoptosis in human colon or breast cancer cell lines and has been reportedly used as a biomarker for breast and bladder cancer [13,14]. Given its versatility, several studies have attempted to utilize taurine as a drug, and notably, the limited derivatization studies yielded effective anticonvulsant, antimicrobial, and anticancer compounds [15,16]. The urea derivative tauromustine exhibits potent in vitro and in vivo antiproliferative activity against several cancer cell lines [17] while the sultam derivative taurolidine was shown to be a clinically useful anticancer agent that did not affect healthy cells (Figure 1) [18]. In addition to its usefulness as a versatile building block for anticancer agents, various taurine‑based amide derivatives have been employed as effective anticonvulsant and antibacterial agents; however, there has been no study evaluating their anticancer activities so far (Figure 2) [19,20]. To date, no extensive structure–activity relationship studies have been conducted to identify specific biological pathways that can accommodate taurine‑based derivatives [21]. This gap in the data prompted us to design and synthesize a group of novel 4-substituted benzoyltaurinamide derivatives and to determine their anticancer activity (Figure 2).

**Figure 3:**
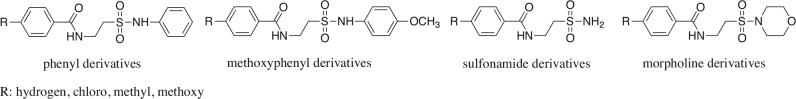
General structure of 4- and N-substituted benzoyltaurinamide derivatives.

In the structure–activity relationship studies conducted using 4-substituted benzoyltaurinamide derivatives, taurine’s sulfonic acid group was functionalized as a primary, secondary, or tertiary sulfonamide that was capable of demonstrating different ionization properties. The resulting compounds were categorized as phenyl, methoxyphenyl, sulfonamide, and morpholine derivatives based on the functional group located at the original sulfonamide position. Here, the amine moiety was functionalized as an amide with hydrogen-, chloro-, methoxy-, and methyl-substituted benzoic acids, while the sulfonamide groups remained untouched (Figure 3). This derivatization strategy allowed us to systematically study the impact of the sulfonamide and amide functional groups as well as the electronic and steric characteristics of the substituents on the aromatic ring of the benzoyltaurinamide derivatives on the compound’s anticancer activity. The resulting derivatives were evaluated against three cancer cell lines and one normal cell line. The most active compounds were subsequently employed to deduce the molecular mechanisms governing the anticancer activity of this class of compounds.

## 2. Results and discussion

### 2.1. Synthesis and characterization of the final compounds

Herein, sixteen 4-substituted benzoyltaurinamide derivatives **(1-16)** were produced via a 5-step reaction scheme (Scheme), and their structures were identified using spectroscopic (1H and 13C NMR, ESI-MS, ATR u1e79) and elemental analytical methods. Briefly, taurine, phthalic anhydride, and potassium acetate were refluxed in an acetic acid solution to generate the 2-phthalimido-ethanesulfonic acid potassium salt, **A** , which was subsequently chlorinated to yield **B** using thionyl chloride via a previously reported procedure [22–26]. The phenyl and methoxyphenyl derivatives were prepared by treating 2-phtalimido-ethanesulfonyl chloride ( **B** , 1 mmol) in pyridine (5 mL) with aniline and 4-methoxyaniline (1 mmol) at 0–5 °C until the starting material was consumed, as evidenced by TLC analysis. *N* -phenyl-2-phtalimido-ethansulfonamide ( **C1** ) and 2-(1,3-dioxoisoindolin-2-yl)- *N* -(4-methoxyphenyl)ethane-1-sulfonamide ( **C2** ) derivatives were achieved in yields of 12–60% after crystallization from acetic acid:water mixture (1:1) [22,26]. The treatment of **B** with concentrated ammonium hydroxide solution produced 2-(1,3-dioxoisoindolin-2-yl)ethane-1-sulfonamide ( **C3** ) in 23% yield, whereas treatment with morpholine and triethylamine in dichloromethane solution yielded 2-(2-(morpholinosulfonyl)ethyl)isoindoline-1,3-dione ( **C4** ) [25]. In the last step, various benzoic acids were treated with 1,1’‑carbonyldiimidazole and diisopropylethylamine in dry acetonitrile, and aqueous solutions of the deprotected derivatives **D1** - **D4** were subsequently added to the respective reaction mixtures. The final compounds (1-16) were achieved in yields of 22–83%, as described in the experimental section. All compounds were characterized via 1H and 13C NMR, ESI–MS, ATR u1e7c, and u1e7d analysis. All spectral data obtained were consistent with the proposed structures (see the experimental section for details).

**Scheme:**
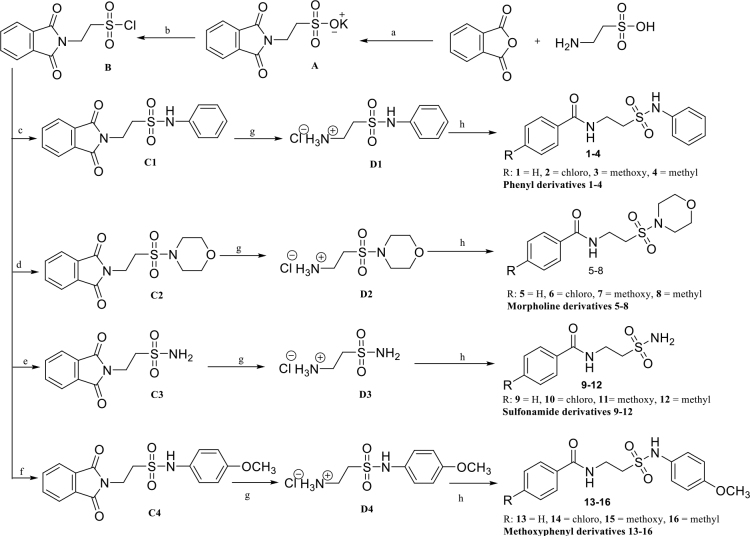
General synthesis of benzoyltaurinamides (1–16). Reagents and conditions: (a) taurine, anhydrous potassium acetate, acetic acid, phthalic anhydride refluxed. (b) A, Phosphorus pentachloride, benzene, reflux. (c) B, aniline, dry pyridine room temperature. (d) B, morpholine, TEA, DCM, 0 °C and then room temperature. (e) B, concentrated ammonium hydroxide solution 0°C and then room temperature. (f) B, p-methoxyaniline, dry pyridine, 0°C and then room temperature. (g) C1, C2, C3, C4, 80% hydrazine hydrate, ethanol, refluxed. (h) D1, D2, D3, D4, water, substituted benzoic acids, 1,1’-carbonyldiimidazole (CDI), diisopropylethylamine (DIPEA), dry acetonitrile (ACN), room temperature.

The 1H NMR spectra of the derivatives exhibited phenyl protons between 6.92 and 7.89 ppm. The methylene protons of taurine and morpholine were observed between 3.20 and 3.72 ppm in the aliphatic field as expected. The secondary sulfonamide peaks for the phenyl and methoxyphenyl derivatives appeared between 9.50 and 10.43 ppm as broad singlets or singlets, whereas these peaks were not detected in the morpholine and sulfonamide derivatives. The sulfonamide derivatives exhibited specific signals for the protons of the primary sulfonamide group at 6.90–6.97 ppm. Finally, the proton signals at 8.46–8.88 ppm were verification of the formation of the amide bond. The carbonyl resonances of the final compounds were observed between 166.19 and 167.14 ppm in the 13C NMR spectra. All other aliphatic and aromatic carbons were observed in the expected regions at 34.65–51.76 ppm and 114.40–142.13 ppm, respectively. In the FT-IR spectra, N–H stretching, as well as SO2 symmetric and asymmetric stretching bands, provided confirmation of the products’ structures. All derivatives exhibited characteristic amide I and amide II peaks at 1606–1645 cm–1 and 1504–1548 cm–1, respectively. In the mass spectra (ESI-MS), the [M+1]+ ion peaks were observed for all titled compounds.

### 2.2. Biological activity

#### 2.2.1. Cytotoxic assay

The cytotoxicity of all derivatives was tested against one nontumorigenic cell line (i.e. MCF-10a) and three cancerous (i.e. MDA-MB-231, PANC-1, and SH-SY5Y) cell lines using the colorimetric methyl tetrazolium test (MTT). The IC50 values of the tested compounds are summarized in Table. The MTT assay revealed that the cytotoxicity of the compounds varied depending on the moiety of the derivative and the type of cell lines tested (Table).

**Table:** Table. Cytotoxic activity (IC50, µM) of the final compounds (1–16) against various cancer cell lines and their selectivity index values.

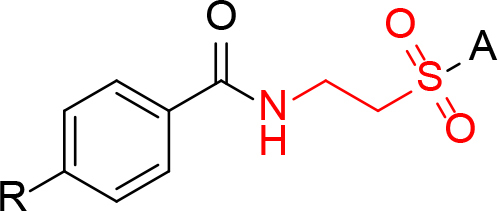							
Com	R	A	SH-SY5Y	PANC-1	MDA-MB-231	MCF-10a	SI*
1	hydrogen	phenyl	92.2 ± 1.5	86.7 ± 1.4	140.4 ± 1.1	78.2 ± 1.6	0
2	chloro	phenyl	29.0 ± 1.5	86.6 ± 1.6	99.7 ± 1.1	232.9 ± 2.1	2.3
3	methoxy	phenyl	57.4 ± 1.6	103.7 ± 1.6	110.6 ± 1.2	98.9 ± 1.2	0
4	methyl	phenyl	75.9 ± 1.6	86.6 ± 1.4	89.7 ± 1.5	132.2 ± 1.7	1.5
5	hydrogen	morpholine	118.4 ± 1.8	24.2 ± 2.1	102.9 ± 1.7	89.6 ± 1.5	0
6	chloro	morpholine	80.6 ± 1.9	8.8 ± 2.1	136.5 ± 1.5	70.7 ± 1.8	0
7	methoxy	morpholine	134.0 ± 1.7	1.2 ± 1.8	77.01 ± 1.7	118.5 ± 1.2	1.5
8	methyl	morpholine	79.3 ± 1.6	120.3 ± 1.2	81.5 ± 1.2	81.4 ± 1.3	1
9	hydrogen	hydrogen	81.1 ± 2.1	85.5 ± 1.7	99.8 ± 1.7	108.8 ± 1.1	1.1
10	chloro	hydrogen	38.6 ± 1.6	89.1 ± 1.7	79.1 ± 1.3	172.1 ± 1.4	2.2
11	methoxy	hydrogen	237.8 ± 1.7	83.8 ± 1.5	114.3 ± 1.3	81.1 ± 1.4	0
12	methyl	hydrogen	79.5 ± 1.8	44.6 ± 1.9	81.0 ± 1.6	106.3 ± 1.4	1.3
13	hydrogen	4-methoxyphenyl	47.5 ± 1.5	17.9 ± 2.1	76.1 ± 1.6	217.8 ± 1.3	2.9
14	chloro	4-methoxyphenyl	26.0 ± 1.4	22.5 ± 1.7	19.9 ± 1.7	109.2 ± 1.2	5.5
15	methoxy	4-methoxyphenyl	39.9 ± 1.6	18.1 ± 1.6	33.9 ± 1.8	118.4 ± 1.4	3.5
16	methyl	4-methoxyphenyl	32.7 ± 1.7	21.4 ± 1.7	15.7 ± 1.9	132.9 ± 1.2	8.5
Cisp			NT	0.017	0.017	0.017	

*Selectivity index represents the ratio between the IC50 value calculated for the cancerous breast cell line (MDA-MB-231) and the IC50 value calculated for the normal breast cell line (MCF-10a). SH-SY5Y–human neuroblastoma; MDA-MB-231–triple-negative breast cancer; PANC‑1–human pancreatic cancer; and MCF-10a–human mammary cell lines.

The final compounds exhibited moderate to good cytotoxicity against PANC-1 cell lines, with IC50 values in the range of 1.2–120.3 µM. Compound 7, a morpholine derivative containing a 4-methoxyphenyl group on the amide functionality, was the most promising product obtained, with an IC50 of 1.2 µM. The other morpholine derivatives, namely, 6 (chloro‑), 5 (hydrogen‑), and 8 (methyl‑substituted) exhibited 8, 24, and 120 times lower activity than 7 (methoxy‑substituted), respectively, thereby proving that methyl substitution diminished the compound’s anticancer activity. The IC50 values of the morpholine derivatives (i.e. 5 and 6) were significantly lower than their sulfonamide (9-12) and phenyl (1-4) counterparts. Similarly, replacing the morpholine group in 7 with phenyl (3), methoxyphenyl (15), or hydrogen (11) moieties reduced the anticancer activity of the compound by 100-, 18-, and 80-fold, respectively. The methoxyphenyl derivatives (13-16) displayed good antiproliferative activity, with IC50 values in the range of 17.9–22.5 µM. From these results, it can be concluded that both morpholine and methoxyphenyl substitution at the sulfonamide group generally increased the compound’s antiproliferative activity against the PANC-1 cell line. In contrast, the ionization properties of the sulfonamide linker did not appear to have any impact on the compound’s activity.

The derivatives also demonstrated well to moderate cytotoxicity against the brain cancer cell line (SH-SY5Y), with IC50 values in the range of 26.0–237.8 µM. The lowest IC50 value was obtained with the phenyl derivative (2). For the phenyl, methoxyphenyl, and sulfonamide derivatives, a chloro-substitution of the amide group produced more potent compounds when compared to the results obtained for their hydrogen‑, methoxy‑, and methyl‑substituted counterparts, with the only exceptions to this being the morpholine derivatives. Interestingly, replacing the phenyl, morpholine, or sulfonamide hydrogen with a methoxyphenyl ring lowered the IC50 values. These results indicated that the ionization properties of the sulfonamide group and chloro‑substitution on the benzamide functionality significantly affected the cytotoxicity of these derivatives against the SH-SY5Y cell line.

The final compounds also exhibited excellent cytotoxicity against the nontumorigenic breast epithelial (MCF-10a) cell line, with IC50 values in the range of 70.7–217.8 µM. Compounds 1-13 displayed moderate IC50 activity (i.e. 77.1–140.4 µM), whereas 14, 15, and 16 (i.e. the methoxyphenyl derivatives) showed good activity against the breast cancer cell line (MDA-MB-231), with IC50 values of 19.9, 33.9, and 15.7 µM, respectively. We noted that all the tested cancerous cell lines were susceptible to the methoxyphenyl derivatives, indicating that the sulfonamide functionality significantly influenced cytotoxicity.

Despite their effectiveness against cancerous cells and healthy cells that exhibit abnormally rapid proliferation, one persistent issue associated with conventional anticancer agents is the severe side effects. Given this, researchers have focused on developing therapeutic agents that exhibit selective cytotoxicity against cancerous cells. Thus, the safety profile of the titled compounds was determined by calculating their selectivity index (SI) values using the formula:

SI = IC50 value calculated for the cancerous breast cell lines (MDA-MB-231) / IC50 value calculated for the normal breast cell line MCF-10a.

Interestingly, compounds 13, 14, 15, and 16 exhibited high selectivity, with SI values of 3, 6, 4, and 9, respectively, for the cancerous when compared to the noncancerous breast cell lines.

The substitution of the phenyl ring in the benzamide portion of the molecule with an electron‑withdrawing chloro-substituent improved the compound’s activity against the SH-SY5Y cell line only, whereas the substitution of phenyl ring with electron‑donating groups did not result in any significantly notable differences in activity against the PANC-1 and MDA-MB-231 cell lines. It was theorized that the derivatization of the sulfonamide part of the molecule was largely responsible for the compounds’ cytotoxicity. We noted that the phenyl and sulfonamide derivatives showed moderate activity against all tested cell lines. Replacing the primary sulfonamide with a secondary sulfonamide containing a phenyl ring did not significantly impact the compound’s cytotoxicity. The presence of heteroatoms increased the contact points in biologically active sites. Since morpholine and methoxyphenyl substitution were known to introduce heteroatoms into the taurine derivatives, their activity was expected to be higher than their counterparts. For our compounds, the morpholine‑substituted derivatives exhibited excellent cytotoxicity against the PANC-1 cell line only, whereas the 4-methoxyphenyl substituent enhanced cytotoxicity against all tested cell lines and was preferentially selective for the breast cancer cell line over the noncancerous breast cell line. This result indicated that the cytotoxicity and selectivity of taurine derivatives could be significantly improved by the incorporation of a substituted aromatic ring in the sulfonamide section of taurine.

#### 2.2.2. The expression levels of proteins related to apoptotic pathway and the rate of apoptosis

Cell death may occur via either necrosis or apoptosis. Necrosis causes inflammation of normal, healthy tissues, whereas apoptotic death is associated with minimal damage to the surrounding cells or tissue, a feature that is highly desired in new anticancer agents. In this study, the mechanism (i.e. via necrosis or apoptosis) triggered by the presence of the taurine derivatives was determined by subjecting compounds 7, 14, and 15 to Annexin V-FITC/PI via flow cytometry. Here, the rates of cell death for each experimental group were classified into three categories: “early apoptotic” in which a positive result was obtained for phosphatidylserine (PS), but a negative result was noted for PI, “late apoptotic” in which a positive result was obtained for both PS and PI, and “necrotic” in which a negative result was seen for PS, but a positive result was noted for PI [27]. We noted that the mitochondria-mediated intrinsic apoptotic pathway was controlled by proapoptotic (BAX) and antiapoptotic (Bcl-2 and Bcl-xL) Bcl-2 proteins. Stimulation of these proteins, therefore, resulted in changes in the mitochondrial membrane’s potential, which triggered the release of mitochondrial cytochrome c into the cytoplasm and activated both caspase-9 and caspase-3 proteins. This, in turn, initiated apoptosis. Both the intrinsic and extrinsic pathways converged on caspase-3 activation, whereas the initiator caspase-9 protein was activated only via the intrinsic pathway. Therefore, the activation of caspase-3 and caspase-9 was indicative of apoptosis via the intrinsic pathway [28].

Emerging evidence suggested that alterations in the expression of antiapoptotic and proapoptotic Bcl-2 proteins were indicators for the presence of cancer cells. Moreover, these proteins were believed to be responsible for the development of resistance to apoptotic stimuli, chemotherapy, and radiation that have recently been noted in cancer cells. Interestingly, a group of Bcl-2 protein inhibitors were reported as potential anticancer agents and has been adopted for clinical use. Therefore, the inhibition of one or more of these proteins has become an attractive anticancer target for researchers tackling apoptosis resistance in tumor cells [29].

**Figure 4:**
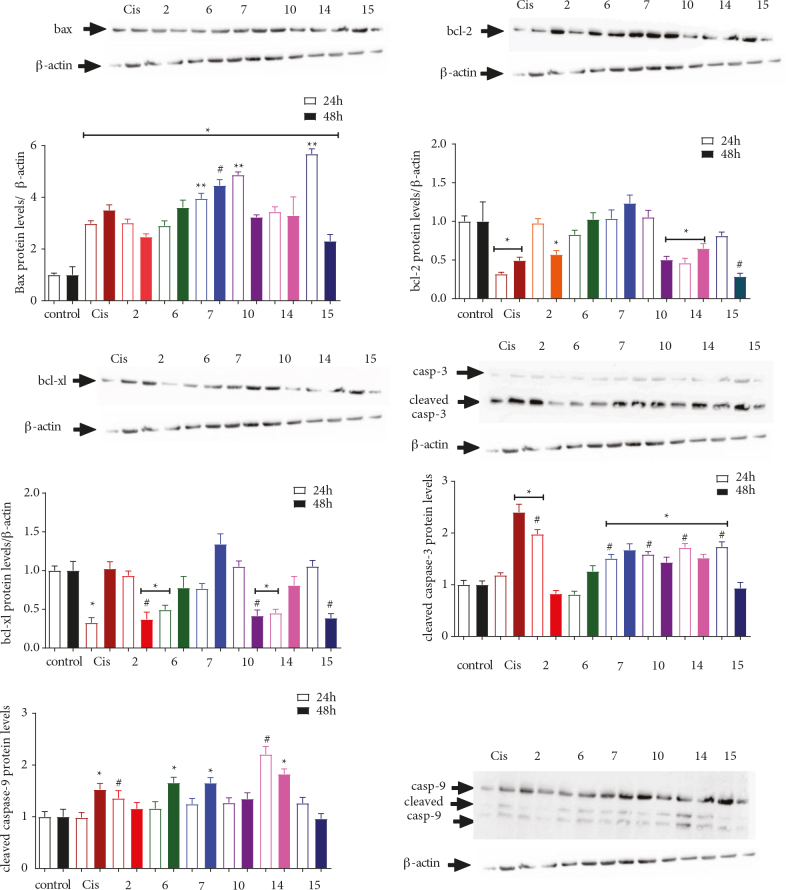
The effect of 2, 6, 7, 10, 14, and 15 on the expression of Bcl-2 and caspase proteins in the PANC-1 cell line. The cells were treated with the previously determined IC50 values of each compound for 24 and 48 h, followed by extraction of the proteins and Western Blot analysis using antibodies against BAX, Bcl-2, Bcl-xL, caspase-3, and caspase-9. β-actin was used as an internal control. The data obtained were expressed as mean ± SD (n = 3). * represented P < 0.05 relative to the control group, whereas # represented P ≤ 0.05 relative to the reference drug cisplatin.

**Figure 5:**
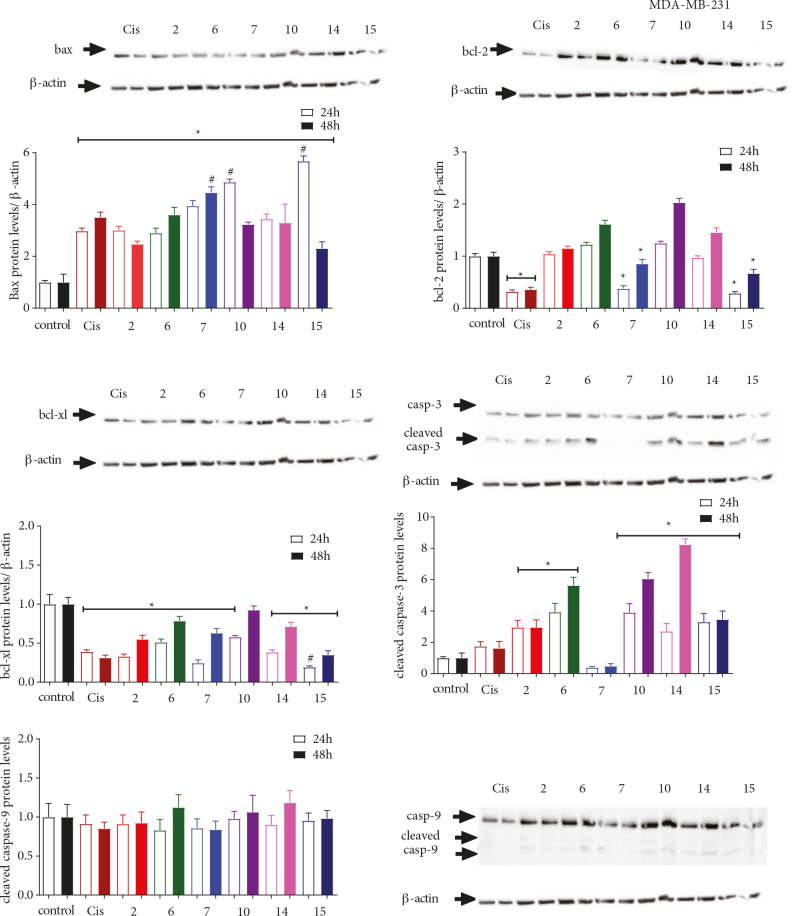
The effects of 2, 6, 7, 10, 14, and 15 on the expression of Bcl-2 and caspase proteins in the MDA-MB-231 (triple-negative; ER, PR, and HER2 negative) cell line. The cells were treated with the previously determined IC50 values of each compound for 24 and 48 h, followed by extraction of the proteins and Western Blot analysis using antibodies against BAX, Bcl-2, Bcl-xL, caspase-3, and caspase-9. β-actin was used as an internal control. The data obtained were expressed as mean ± SD (n = 3). * represents P < 0.05 relative to the control group, whereas # represents P ≤ 0.05 relative to the reference drug cisplatin.

**Figure 6:**
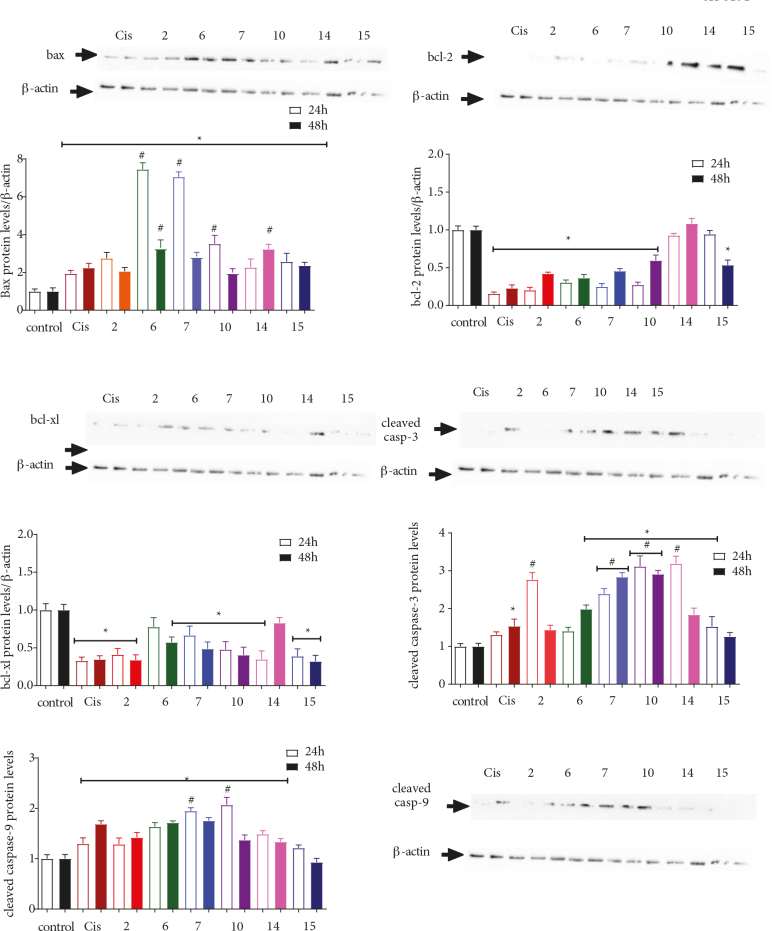
The effects of 2, 6, 7, 10, 14, and 15 on the expression of Bcl-2 and caspase proteins in the SH-SY5Y cell line. The cells were treated with the previously determined IC50 values of each compound for 24 and 48 h, followed by extraction of the proteins and Western Blot analysis using antibodies against BAX, Bcl-2, Bcl-xL, caspase-3, and caspase-9. β-actin was used as an internal control. The data obtained were expressed as mean ± SD (n = 3). * represents P < 0.05 relative to the control group, whereas # represents P ≤ 0.05 relative to the reference drug cisplatin.

In light of these findings, the extent of protein expression, namely, BAX, Bcl-2, Bcl-xL, cleaved caspase-3, and caspase-9, in the apoptotic pathway was examined to determine the mechanism through which cellular apoptosis was induced. Compounds 2, 6, 7, 10, 14, and 15 were chosen for further evaluation, and the PANC-1, SH-SY5Y, and MDA-MB-231 cell lines were treated with the determined cytotoxic concentrations of the selected compounds for 24 h and 48 h (Figures 4–6).

**Figure 7:**
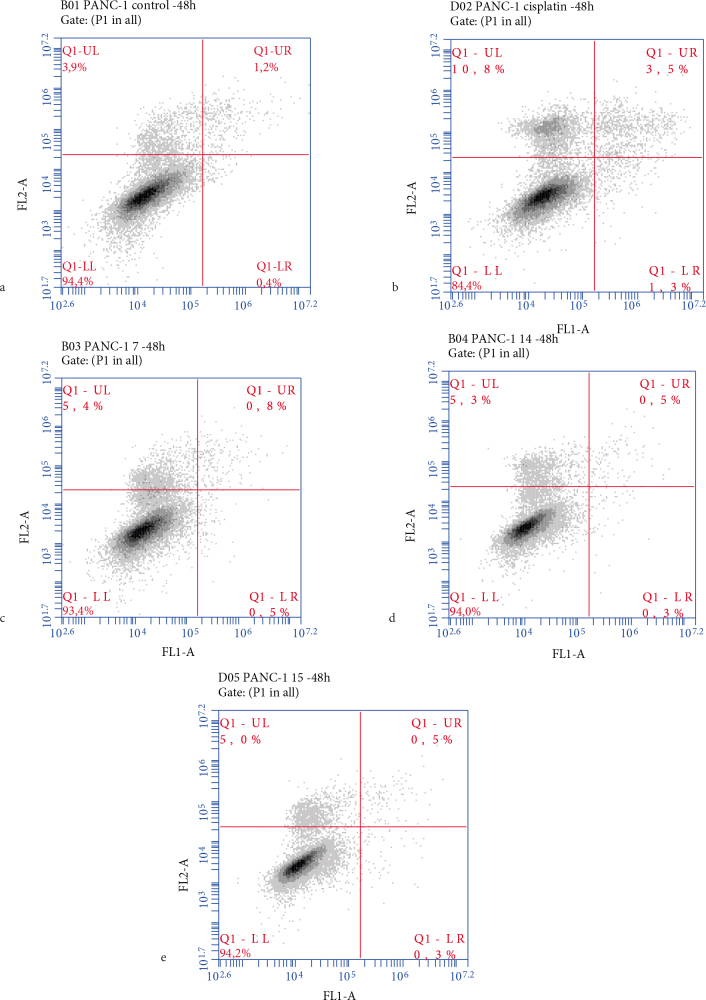
Apoptotic assay via flow cytometry. The PANC-1 cells were treated with 7, 14, and 15 at their calculated IC50 values for 48 h. The cells were stained with Annexin V-FITC/PI and then subjected to flow cytometry. The rate of apoptosis of diverse groups of PANC-1 cells (a) the control group, (b) cisplatin, (c) 7, (d) 14, and (e) 15.

Here, Western Blot analysis revealed that compound 14 (i.e. the chloro‑substituted methoxyphenyl derivative) was the only molecule that increased BAX, caspase-3, and caspase-9 expression levels, whereas there was a decrease in the expression of Bcl-2 and Bcl-xL in the PANC-1 cell line after 24 and 48 h of exposure (Figure 4). Replacing the chloro group (i.e. the methoxy derivative 15) resulted in the opposite effect on the expression of Bcl-xL after 24 h, and caspase‑3 and caspase‑9 after 48 h. Compound 7 (i.e. the methoxy‑substituted morpholine derivative) was the most cytotoxic molecule tested, with an IC50 value of 1.2 µM against the PANC-1 cell line; we noted that 7 effectively upregulated the expression of BAX, caspase-3, and caspase-9. Conversely, compound 7 did not show any effect on the expression of Bcl-2 and seemed to slightly decreased Bcl-xL levels after only 24 h. Thus, it would seem that substituting the methoxyphenyl moiety (i.e. 15) with a morpholine group retarded Bcl-2 expression while potentiating the impact on the caspase‑3 and caspase‑9 levels. Interestingly, 2 (i.e. the chloro‑substituted phenyl derivative) possessed a high IC50 value of 86.64 µM, and significantly upregulated the expression of BAX after 24 h and Bcl-2 after 48 h when compared to chloro‑substituted compounds such as 6, 10, and 14 (i.e. IC50 values of 8.8, 89.1, and 22.5 µM, respectively). Except for the methoxy‑substituted methoxyphenyl 15, all compounds triggered a notable increase in the expression of caspase-9. In contrast, only 7 (i.e. the methoxy‑substituted morpholine derivative), 10 (i.e. the chloro‑substituted sulfonamide derivative), and 14 (i.e. the chloro‑substituted methoxyphenyl derivative) effectively increased the expression of caspase-3 after 24 and 48 h. Except for 6, all compounds containing the chloro‑substituted morpholine derivative increased caspase‑3 expression better than the reference drug cisplatin after 24 h. The effects of 2 and 6 on the expression of caspase‑9 levels, 7 on the BAX levels, 15 on the Bcl-2 levels, and compounds 2, 10, and 15 on the expression of Bcl‑xL were found to be better than the expression observed with the reference drug cisplatin after 48 h. Annexin V-FITC/PI experiments have revealed that treatment of the cell lines with 7, 14, and 15 at their cytotoxic concentrations for 48 h resulted in no significant changes in the apoptotic cell ratios (Figure 7). Despite the impact exerted by these molecules on apoptotic proteins, Annexin V-FITC/PI results showed that there might be another mechanism at play that may control the cytotoxicity of these compounds. Further study on this topic is required.

**Figure 8:**
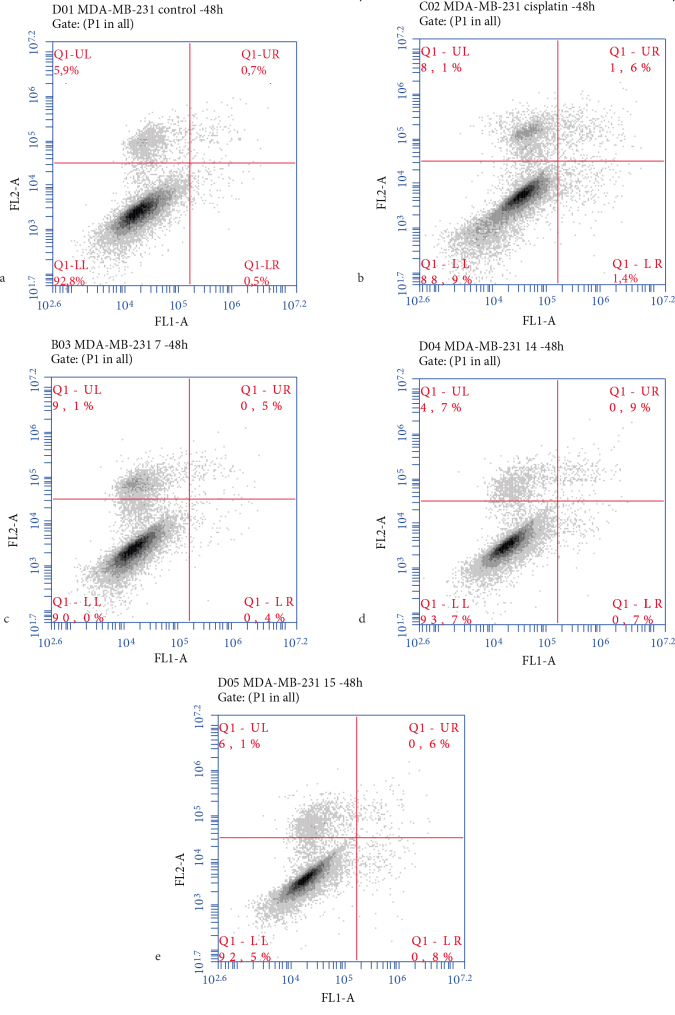
Apoptotic assay via flow cytometry. The MDA-MB-231 cells were treated with 7, 14, and 15 at their calculated IC50 values for 48 h. The cells were stained with Annexin V-FITC/PI and subsequently subjected to flow cytometry. The rate of apoptosis of various groups of MDA-MB-231 cells in (a) the control group, (b) cisplatin, (c) 7, (d) 14, and (e) 15.

Treating the MDA-MB-231 cell lines with compounds 2, 6, 7, 10, 14, and 15, which had IC50 values of 99.7, 79.1, 136.5, 77, 19.9, and 33.9 µM, respectively, significantly increased BAX protein levels after only 24 h, whereas the expression of Bcl-xL decreased by 2.08- to 4.55-fold after 24 h and by 2- to 5-fold after 48 h when compared to the control samples (Figure 5). Interestingly, only 7 (i.e. the methoxy‑substituted morpholine derivative) and 15 (i.e. the methoxy‑substituted methoxyphenyl derivative) decreased the expression of Bcl-2 by 2- to 3-fold when compared to the control sample after 24 and 48 h. Conversely, only 6 (i.e. the chloro‑substituted morpholine derivative), 10 (i.e. the chloro‑substituted sulfonamide derivative), and 14 (i.e. the chloro‑substituted methoxyphenyl derivative) slightly increased caspase-9 activity by 1.06-, 1.12-, and 1.18-fold relative to the control after only 48 h. Except for 7, all compounds were shown to improve caspase-3 expression levels by 3.96- to 8.2-fold relative to the control group. Interestingly, while 7 and 10 exhibited almost the same cytotoxicity (i.e. IC50 values of 79 and 77 µM, respectively) against the MDA-MB-231 cell line, their influence on the caspase‑3 levels differed by almost 12-fold. The effects exerted by 7, 10, and 15 on BAX expression and by compound 15 on Bcl-xL expression were statistically higher than the results associated with the reference drug. Annexin V-FITC/PI results have revealed that 14 and 15 slightly increased the percent of the positive apoptotic cells from 1.2% to 1.6% and 1.4%, respectively, even though compound 7 exhibited no changes (Figure 8). Given these results, it was theorized that 7 exerted its influence via a caspase-independent mechanism, as evidenced by the lack of change associated with the expression of caspase‑3 in the presence of this compound. Interestingly, replacing the chloro group (i.e. 14) with a methoxy group (i.e. 15) resulted in a change in the anticancer mechanistic pathway of the methoxyphenyl derivatives, as 14 appeared to follow the intrinsic apoptotic pathway; on the other hand, 15 did not exert any effect on the caspase-9 expression levels but increased the expression of caspase-3. Therefore, it was proposed that 15 induced apoptosis via a different caspase-dependent pathway [30].

**Figure 9:**
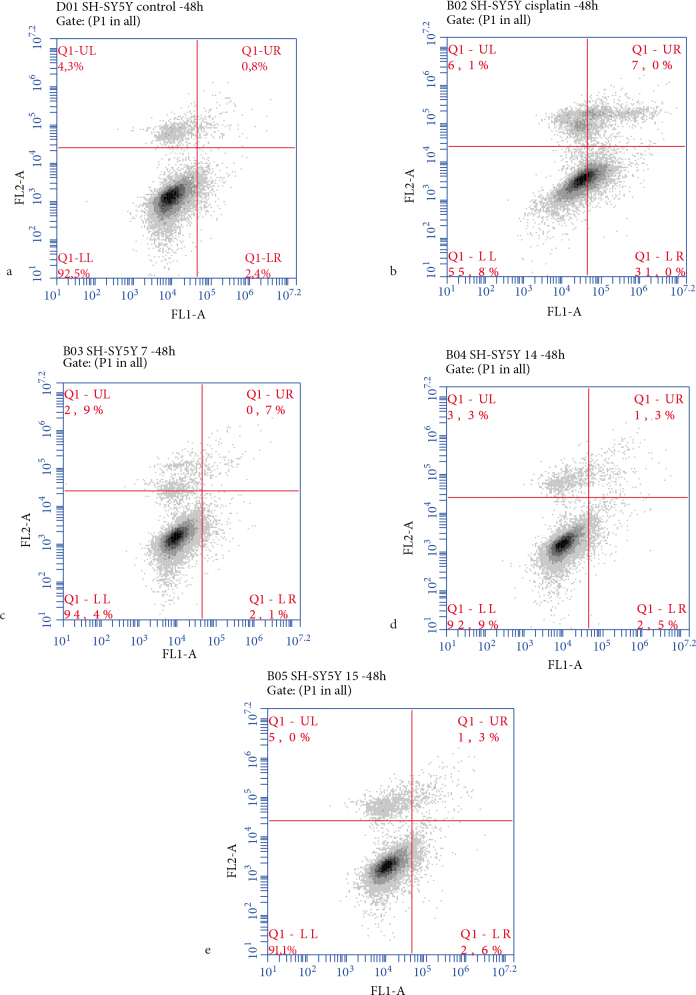
Apoptosis assay via flow cytometry. The SH-SY5Y cells were treated with 7, 14, and 15 at their calculated IC50 values for 48 h. The cells were stained with Annexin V-FITC/PI and subsequently subjected to flow cytometry. The rate of apoptosis of various groups of SH-SY5Y cells in (a) the control group, (b) cisplatin, (c) 7, (d) 14, and (e) 15.

As noted, compounds 2, 6, 7, 10, 14, and 15 exerted their influence by increasing BAX (1.740- to 7.5-fold), caspase-3 (1.259- to 3.191-fold relative to the control), caspase-9 (1.214- to ­2.068-fold relative to the control), while decreasing Bcl-2 (1.68- to 4.9-fold for all except 14) and Bcl-xL (1.288- to 3.125-fold relative to the control) expression levels in the SH-SY5Y cell line (Figure 6). Interestingly, while 6 and 7 were the least cytotoxic compounds in the SH-SY5Y cell line with IC50 values of 80.62 and 134 µM, respectively, there were uncanny similarities in their activity values and protein expression levels when compared to the other compounds. Compounds 6 (i.e. the chloro‑substituted morpholine derivative), 7 (i.e. the methoxy‑substituted morpholine derivative), and 10 (i.e. the chloro‑substituted sulfonamide derivative) influenced the expression of BAX. Compounds 2 (i.e. the chloro‑substituted phenyl derivative), 7, 10, and 14 influenced the caspase-3 expression levels, whereas the expression of caspase‑9 was affected by the presence of 7 and 10. In these cases, the expression levels of the respective proteins were better when compared to the standard drug cisplatin. Compound 7 gave no positive results when subjected to Annexin V-FITC/PI experiments, whereas apoptotic cell ratios were slightly increased from 3.2% to 3.8% and 3.9%, respectively, after the application of 14 and 15 (Figure 9). According to these results, 14 and 15 acted through the intrinsic apoptotic pathway, whereas the cytotoxicity of 7 was supported by other cell death mechanisms.

## 3. Conclusion

In this study, sixteen new 4-substituted benzoyltaurinamide derivatives were synthesized, and their anticancer activity was investigated using one normal (i.e. MCF-10a) and three cancerous cell lines (i.e. MDA-MB-231, PANC-1, and SH-SY5Y) via MTT assay. The mechanism through which this group of compounds exerted their cytotoxic activity was determined using the most active compounds. Here, the expression levels of BAX, Bcl-2, and Bcl-xL, as well as cleaved caspase-3 and caspase-9, were examined via Western Blot assay, and apoptosis was determined using the Annexin V-FITC/PI test in combination with flow cytometry.

Of the phenyl **(1-4)** and sulfonamide **(9-12)** derivatives prepared in this study, only the chloro‑bearing compounds 2 and 10 exhibited good cytotoxicity with IC50 values of 29.0 and 38.6 µM, respectively. These compounds were preferentially selective against SH-SYH cells when compared to the other cell lines. All morpholine derivatives (5-8), except 8, exhibited low IC50 values (i.e. 24.2, 8.8, and 1.2 µM) against the PANC-1 cell line only. In contrast, the methoxyphenyl derivatives (13-16) were effective against all tested cell lines, with low cytotoxic doses between 21.4 and 47.5 µM relative to their counterparts. Moreover, these compounds exhibited preferential selectivity toward cancerous breast cells than noncancerous cells, as evidenced by SI values between 3 and 9. Western Blot analysis revealed that all compounds, except for 7, induced the intrinsic apoptotic pathway by upregulating BAX, caspase-3, and caspase-9, while downregulating Bcl-2 and Bcl-xL expression levels in PANC-1 cell line either after 24 or 48 h. The same trend was noted in the SH-SY5Y cell line for all compounds after 24 and 48 h. For the MDA-MB-231 cell line, only 14 seemed to alter the intrinsic pathway-related expression of protein at the 24 or 48 h time mark. Annexin V-FITC/PI test results revealed that 7, 14, and 15 followed different cell death pathways in the MDA-MB‑231 cell line. These mechanisms can be categorized as: i) a caspase-dependent apoptotic pathway that includes the upregulation of BAX, caspase-3, and caspase-9 along with the downregulation of Bcl-2 and Bcl-xL expression, ii) a caspase-independent mechanism, and iii) a caspase-dependent apoptotic pathway that excludes caspase-9 activation. Even though the Annexin V-FITC/PI test results were negative for the PANC-1 cell lines for compounds 7, 14, and 15, the intrinsic pathway-related expression of the protein was altered as expected. Therefore, the anticancer effects of these compounds seem to support evidence of other cell death mechanisms. The same mechanism was detected for 7 in the SH-SYH cell lines, even though this cell line was subjected to the intrinsic pathway in the presence of compounds 14 and 15. Compounds 14 and 15 deserve further in vivo studies as they comprise an underexplored taurine structure compared to the classical anticancer drugs which already have resistance problems. As a result, this study has shown that modifying both the sulfonic acid and amine groups of taurine influences the selectivity, cytotoxicity, and the mechanism of cell death, thereby offering valuable insight into the design of new taurine-based anticancer derivatives.

## 4. Experimental

### 4.1. Chemistry

Phthalic anhydride, potassium acetate, aniline, 4-methylbenzoic acid, and 4-chloro benzoic acid were purchased from Merck (Germany). All the other reagents, reactants, and anhydrous solvents were purchased from Sigma-Aldrich (Germany), Carlo Erba (France), and Interlab (Germany). All reactions involving air- or moisture-sensitive compounds were performed under a nitrogen atmosphere using dried glassware and syringes to transfer solutions. 1H NMR spectra were recorded on a Varian AS 400 Mercury Plus NMR spectrometer using DMSO- *d* *6* as a solvent. Chemical shifts were reported in parts per million (ppm), and the coupling constants ( *J* ) were expressed in hertz (Hz). Splitting patterns were designated as follows: *s* , singlet; *d* , doublet; *t* , triplet; *m* , multiplet; *brs* , broad singlet. Infrared spectra were run on a Perkin Elmer Spectrum 100 FT-IR equipped with a Universal ATR Sampling Accessory, and the frequencies were expressed in cm–1. Analytical thin-layer chromatography (TLC) was conducted on Merck silica gel F-254 plates. Flash chromatography purifications were performed on Merck silica gel 60 (230–400 mesh ASTM) as the stationary phase. Silica gel was packed in with 2 cm diameter glass column to a 6 cm height bed with a solvent mixture consisting of ethyl acetate and hexane. Sample was loaded onto the column and eluted with a mobile phase of the aforementioned mixture. Melting points were taken with a Stuart® (SMP30) melting point apparatus in open capillary tubes and were uncorrected. The solvents used in MS measurements were ethanol, acetonitrile, and water (LC-MS grade), purchased from Sigma-Aldrich (Germany). The ESI-MS (electrospray ionization-mass spectrophotometry) spectra were obtained using a Thermo Scientific DSQ II (Austin, TX USA) equipped with an electrospray source (ESI) operating in both positive and negative ions. Raw data were collected and processed by XcaliburTM 2.0 software. Elemental analyses (C, H, N, and S) were performed using a Leco TruSpec CHNS Microanalyzer (Leco Corporation, St. Joseph, MI, USA), and results were within ±0.4% of calculated values.

#### 4.1.1. General procedure a: synthesis of D1, D2, D3, D4

2-Amino- *N* -phenylethane-1-sulfonamide.HCl ( **D1** ) and 2-(morpholinosulfonyl) ethan-1-amine.HCl ( **D2** ) were prepared according to the procedure that was described before [25].

The starting compounds 2-(1,3-dioxoisoindolin-2-yl)ethane-1-sulfonamide ( **C3** ) and 2-(1,3-dioxoisoindolin-2-yl)- *N* -(4-methoxyphenyl)ethane-1-sulfonamide ( **C4** ) were synthesized as previously reported [26]. To a 2.8 mmol solution of compound **C3** or **C4** in 10 mL ethanol, hydrazine hydrate solution (7.0 mmol) was added and refluxed. After the consumption of the starting compounds, concentrated HCl (7.5 mL) was added and stirred for 1 h at room temperature. The mixture was filtered, and the solvent evaporated under vacuum. The crude product was triturated with dichloromethane/diethyl ether to produce 2-aminoethane-1-sulfonamide.HCl ( **D3** ) and triturated with ethanol/petroleum ether to produce 2-amino- *N* -(4-methoxyphenyl)ethane-1-sulfonamide.HCl ( **D4** ) as a white solid.

#### 4.1.2. General procedure B: synthesis of compounds 1-16

Various benzoic acids (1.0 mmol), 1-1’-carbonyldiimidazole (CDI, 1.0 mmol), and diisopropylethylamine (DIPEA, 2.0 mmol) were dissolved in dry acetonitrile (ACN, 8.0 ml) stirred for ½ h at room temperature. Hydrolyzed derivatives (1.0 mmol) **D1** , **D2** , **D3** , and **D4** were dissolved in 10 ml of water added to the reaction mixture. After the consumption of the starting material, the solvent was evaporated. The crude product was purified by silica column chromatography, and the obtained solid was crystallized with an appropriate solvent to yield 4-substituted- *N* -(2-( *N* -phenylsulfamoyl)ethyl)benzamide (1-4), 4-substituted-N-(2-(morpholinosulfonyl)ethyl)benzamide derivatives (5-8), 4-substituted-N-(2-sulfamoylethyl)benzamide (9-12), and 4-substituted-N-(2-(N-(4-methoxyphenyl)sulfamoyl)ethyl)benzamide derivatives (13-16) as white solid [31,32]. Compound 9 was reported previously for its anticonvulsant activity [15,33–36]. For compounds 1-13 registered CAS numbers have been assigned previously. However, their synthesis procedures, chemical properties, and structural characterization are not available in the literature.

##### 4.1.2.1. N-(2-(N-phenylsulfamoyl)ethyl)benzamide (1): CAS: 1326627-91-1.

A solution of 2-amino- *N* -phenylethane-1-sulfonamide.HCl (D1, 1.0 mmol) in water (10 mL) was treated with a solution of benzoic acid (1.0 mmol), CDI (1.0 mmol), and DIPEA (2.0 mmol) in dry ACN (8.0 mL) according to the general procedure B. The crude product was purified by silica gel column chromatography eluting with ethyl acetate:hexane (1:1) and crystallized from isopropanol to afford the title compound 1 as a white solid (0.134 g, 44%); *R* *f* = 0.36 (ethyl acetate:hexane 1:1); m.p. 123.7–124.7 °C; 1H NMR (400 MHz, DMSO- *d* 6) δ: 3.38–3.45 (m, 2H, Aliph-H), 3.66–3.71 (m, 2H, Aliph-H), 7.12–7.16 (m, 1H, Ar-H), 7.25–7.28 ( m, 2H, Ar-H), 7.33–7.37 (m, 2H, Ar-H), 7.47–7.50 (m, 2H, Ar-H), 7.54–7.58 (m, 1H, Ar-H), 7.80–7.83 (m, 2H, Ar-H), 8.59–8.61 (m, 1H, -CONH-), 9.88 (brs, 1H, -SO2NH-). 13C NMR (100 MHz, DMSO- *d* 6) δ: 35.1 (Aliph-C), 50.7 (Aliph-C), 120.9 (2 × Ar-C), 124.9 (Ar-C), 128.0 (2 × Ar-C), 129.2 (2 × Ar-C), 130.2 (2 × Ar-C), 132.2 (Ar-C), 134.8 (Ar-C), 138.9 (Ar-C), 167.3 (C=O). FT-IR υmaks (cm–1): 3411 (N-H stretching), 1645 (amide I band), 1538 (amide II band), 1312 (SO2 asymmetric stretching), 1142 (SO2 symmetric stretching) cm–1. Exact mass: 304.09, MS (EI): *m/z* (%); 305 (23, M+1), 105 (100). Elemental analysis calculated (%) for C15H16N2O3S: C, 59.19; H, 5.30; N, 9.20; S, 10.54. Found: C, 58.91; H, 5.14; N, 9.09; S, 10.75.

##### 4.1.2.2. 4-Chloro-N-(2-(N-phenylsulfamoyl)ethyl)benzamide (2): CAS: 1326627-94-4

A solution of 2-amino- *N* -phenylethane-1-sulfonamide.HCl ( **D1** , 1.0 mmol) in water (10 mL) was treated with a solution of 4-chlorobenzoic acid (1.0 mmol), CDI (1.0 mmol), and DIPEA (2.0 mmol) in dry ACN (8.0 mL) according to the general procedure B. The crude product was purified by silica gel column chromatography eluting with ethyl acetate:hexane (1:1) and crystallized from isopropanol:water to afford the title compound 2 as a white solid (0.098 g, 29%); Rf = 0.39 (ethyl acetate:hexane 1:1); m.p. 143.8–144.8 °C; 1H NMR (400 MHz, DMSO-d6) δ: 3.38–3.40 (m, 2H, Aliph-H), 3.64–3.69 (m, 2H, Aliph-H), 7.14 (t, J = 7.3 Hz, 1H, Ar-H), 7.26 (m, J = 7.6 Hz, 2H, Ar-H), 7.33–7.37 (m, 2H, Ar-H), 7.57 (d, J = 8.6 Hz, 2H, Ar-H), 7.83 (d, J = 8.6 Hz, 2H, Ar-H), 8.67-8.70 (m, 1H, -CONH-), 9.86 (brs, 1H, -SO2NH-). 13C NMR (100 MHz, DMSO-d6) δ: 35.2 (Aliph-C), 50.5 (Aliph-C), 120.8 (2 × Ar-C), 124.9 (Ar-C), 129.3 (2 × Ar-C), 129.9 (2 × Ar-C), 130.2 (2 × Ar-C), 133.6 (Ar-C), 137.1 (Ar-C), 138.9 (Ar-C), 166.2 (C=O). FT-IR υmaks (cm–1): 3391 (N-H stretching), 1645 (amide I band), 1541 (amide II band), 1311 (SO2 asymmetric stretching), 1142 (SO2 symmetric stretching) cm–1. Exact mass: 338.05, MS (EI): m/z (%); 339 (1, M+1), 41 (100). Elemental analysis calculated (%) for; C15H15ClN2O3S: C, 53.18; H, 4.46; N, 8.27; S, 9.46. Found: C, 53.15; H, 4.66; N, 8.32; S, 9.23.

##### 4.1.2.3. 4-Methoxy-N-(2-(N-phenylsulfamoyl)ethyl)benzamide (3): CAS: 1327651-78-4.

A solution of 2-amino- *N* -phenylethane-1-sulfonamide.HCl ( **D1** , 1.0 mmol) in water (10 mL) was treated with a solution of 4-methoxybenzoic acid (1.0 mmol), CDI (1.0 mmol), and DIPEA (2.0 mmol) in dry ACN (8.0 ml) according to the general procedure B. The crude product was purified by silica gel column chromatography eluting with ethyl acetate:hexane (2:1) to afford the title compound 3 as a white solid (0.277 g, 83%); Rf = 0.33 (ethyl acetate:hexane 2:1); m.p. 121.3–122.3 °C; 1H NMR (400 MHz, DMSO-d6) δ: 3.35–3.38 (m, 2H, Aliph-H), 3.63–3.68 (m, 2H, Aliph-H), 3.84 (s, 3H, -OCH3), 7.01 (d, J = 7.6 Hz, 2H, Ar-H), 7.12–7.16 (m, 1H, Ar-H), 7.26–7.28 (m, 2H, Ar-H), 7.33–7.37 (m, 2H, Ar-H), 7.79 (d, J = 7.6 Hz, 2H, Ar-H), 8.44–8.47 (m, 1H, -CONH-), 9.87 (brs, 1H, -SO2NH-); 13C NMR (100 MHz, DMSO-d6) δ: 35.1 (Aliph-C), 50.8 (Aliph-C), 56.2 (-OCH3), 114.4 (2 × Ar-C), 120.9 (2 × Ar-C), 124.9 (Ar-C), 127.1 (Ar-C), 129.9 (2 × Ar-C), 130.2 (2 × Ar-C), 138.9 (Ar-C), 162.6 (Ar-C), 166.8 (C=O). FT-IR υmaks (cm–1): 3339, 3258 (N-H stretching), 1632 (amide I band), 1504 (amide II band), 1301 (SO2 asymmetric stretching), 1138 (SO2 symmetric stretching) cm–1. Exact mass: 334.10, MS (EI): m/z (%); 335 (10, M+1), 135 (100). Elemental analysis calculated (%) for C16H18N2O4S: C, 57.47; H, 5.43; N, 8.38; S, 9.59. Found: C, 57.21; H, 5.29; N, 8.19; S, 9.60.

##### 4.1.2.4. 4-Methyl-N-(2-(N-phenylsulfamoyl)ethyl)benzamide (4): CAS: 1328351-06-9.

A solution of 2-amino- *N* -phenylethane-1-sulfonamide.HCl ( **D1** , 1.0 mmol) in water (10 mL) was treated with a solution of 4-methylbenzoic acid (1.0 mmol), CDI (1.0 mmol), and DIPEA (2.0 mmol) in dry ACN (8.0 mL) according to the general procedure B. The crude product was purified by silica gel column chromatography eluting with ethyl acetate:hexane (1:1) and crystallized from isopropanol to afford the title compound 4 as a white solid (0.251 g, 79%); Rf = 0.43 (ethyl acetate:hexane 1:1); m.p. 149.7–150.7 °C; 1H NMR (400 MHz, DMSO-d6) δ: 2.38 (s, 3H, -CH3), 3.36–3.39 (m, 2H, Aliph-H), 3.64–3.67 (m, 2H, Aliph-H), 7.12–7.16 (m, 1H, Ar-H), 7.25–7.30 (m, 4H, Ar-H), 7.33–7.37 (m, 2H, Ar-H), 7.72 (d, J = 8.2 Hz, 2H, Ar-H), 8.50–8.53 (m, 1H, -CONH-), 9.88 (brs, 1H, -SO2NH-). 13C NMR (100 MHz, DMSO-d6) δ: 21.8 (-CH3), 35.1 (Aliph-C), 50.7 (Aliph-C), 120.9 (2 × Ar-C), 124.9 (Ar-C), 128.0 (2 × Ar-C), 129.7 (2 × Ar-C), 130.2 (2 × Ar-C), 132.1 (Ar-C), 138.9 (Ar-C), 142.1 (Ar-C), 167.1 (C=O). FT-IR υmaks (cm–1): 3399 (N-H stretching), 1640 (amide I band), 1542 (amide II band), 1313 (SO2 asymmetric stretching), 1142 (SO2 symmetric stretching) cm–1. Exact mass: 318.10, MS (EI): m/z (%); 319 (23, M+1), 119 (100). Elemental analysis calculated (%) for C16H18N2O3S: C, 60.36; H, 5.70; N, 8.80; S, 10.07. Found: C, 60.37; H, 5.50; N, 8.68; S, 10.18.

##### 4.1.2.5. N-(2-(morpholinosulfonyl)ethyl)benzamide (5): CAS: 1216894-89-1.

A solution of 2-(morpholinosulfonyl)ethan-1-amine.HCl ( **D2** , 1.0 mmol) in water (10 mL) was treated with a solution of benzoic acid (1.0 mmol), CDI (1.0 mmol), and DIPEA (2.0 mmol) in dry ACN (8.0 mL) according to the general procedure B. The crude product was purified by silica gel column chromatography eluting with ethyl acetate:hexane (1:3) to afford the title compound 5 as a white solid (0.119 g, 40%); Rf = 0.13 (ethyl acetate:hexane 2:1); m.p. 107–108 °C; 1H NMR (400 MHz, DMSO-d6) δ: 3.20–3.22 (m, 4H, Aliph-H), 3.36–3.38 (m, 2H, Aliph-H), 3.37–3.70 (m, 6H, Aliph-H), 7.50–7.53 (m, 2H, Ar-H), 7.56–7.60 (m, 1H, Ar-H), 7.86–7.89 (m, 2H, Ar-H), 8.68–8.71 (m, 1H, -CONH-). 13C NMR (100 MHz, DMSO-d6) δ: 34.8 (Aliph-C), 46.2 (2 × Aliph-C), 47.7 (Aliph-C), 66.7 (2 × Aliph-C), 128.0 (2 × Ar-C), 129.2 (2 × Ar-C), 132.3 (Ar-C), 134.9 (Ar-C), 167.2 (C=O). FT-IR υmaks (cm-1): 3328 (N-H stretching), 1639 (amide I band), 1536 (amide II band), 1318 (SO2 asymmetric stretching), 1144 (SO2 symmetric stretching) cm–1. Exact mass: 298.10, MS (EI): m/z (%); 299 (7, M+1), 212 (100). Elemental analysis calculated (%) for C13H18N2O4S: C, 52.33; H, 6.08; N, 9.39; S, 10.75. Found: C, 52.24; H 6.24; N, 9.42; S,10.45 4.1.2.6. 4-Chloro-N-(2-(morpholinosulfonyl)ethyl)benzamide (6): CAS: 1216690-49-1.

A solution of 2-(morpholinosulfonyl)ethan-1-amine.HCl ( **D2** , 1.0 mmol) in water (10 mL) was treated with a solution of 4-chlorobenzoic acid (1.0 mmol), CDI (1.0 mmol), and DIPEA (2.0 mmol) in dry ACN (8.0 mL) according to the general procedure B. The crude product was purified by silica gel column chromatography eluting with ethyl acetate:hexane (1:5) and crystallized from isopropanol:ethanol to afford the title compound 6 as a white solid (0.142 g, 43%); Rf = 0.14 (ethyl acetate:hexane 2:1); m.p. 162–163 °C; 1H NMR (400 MHz, DMSO-d6): δ: 3.20–3.22 (m, 4H, Aliph-H), 3.36–3.38 (m, 2H, Aliph-H), 3.66–3.69 (m, 6H, Aliph-H), 7.59 (d, J = 8.5 Hz, 2H, Ar-H), 7.89 (d, J = 8.6 Hz, 2H, Ar-H), 8.81-8.88 (m, 1H, -CONH-). 13C NMR (100 MHz, DMSO-d6) δ: 34.8 (Aliph-C), 46.2 (2 × Aliph-C), 47.6 (Aliph-C), 66.7 (2 × Aliph-C), 129.4 (2 × Ar-C), 130.0 (2 × Ar-C), 133.7 (Ar-C), 137.1 (Ar-C), 166.2 (C=O). FT-IR υmaks (cm–1): 3341 (N-H stretching), 1635 (amide I band), 1544 (amide II band), 1319 (SO2 asymmetric stretching), 1102 (SO2 symmetric stretching) cm–1. Exact mass: 332.06, MS (EI): m/z (%); 333 (2, M+1), 41 (100). Elemental analysis calculated (%) for; C13H17ClN2O4S: C, 46.92; H, 5.15; N, 8.42; S, 9.63. Found: C, 46.80; H, 5.29; N, 8.12; S, 9.65.

##### 4.1.2.7. 4-Methoxy-N-(2-(morpholinosulfonyl)ethyl)benzamide (7): CAS: 899739-38-9.

A solution of 2-(morpholinosulfonyl)ethan-1-amine.HCl ( **D2** , 1.0 mmol) in water (10 mL) was treated with a solution of 4-methoxybenzoic acid (1.0 mmol), CDI (1.0 mmol), and DIPEA (2.0 mmol) in dry ACN (8.0 mL) according to the general procedure B. The crude product was purified by silica gel column chromatography eluting with ethyl acetate:hexane (1:3) and crystallized from isopropanol:water to afford the title compound 7 as a white solid (0.082 g, 25%); Rf = 0.14 (ethyl acetate:hexane 2:1); m.p. 183.5–184.5 °C; 1H NMR (400 MHz, DMSO-d6): δ: 3.20–3.21 (m, 5H, Aliph-H), 3.66–3.67 (m, 7H, Aliph-H), 3.84 (s, 3H, -OCH3), 7.03–7.05 (m, 2H, Ar-H), 7.84–7.86 (m, 2H, Ar-H), 8.53–8.56 (m, 1H, -CONH-). 13C NMR (100 MHz, DMSO-d6) δ: 34.7 (Aliph-C), 46.2 (2 × Aliph-C), 47.8 (Aliph-C), 56.2 (-OCH3), 66.7 (2 × Aliph-C), 114.4 (2 × Ar-C), 127.2 (Ar-C), 129.9 (2 × Ar-C), 162.6 (Ar-C), 166.7 (C=O). FT-IR υmaks (cm-1): 3290 (N-H stretching), 1605 (amide I band), 1543 (amide II band), 1332 (SO2 asymmetric stretching), 1111 (SO2 symmetric stretching) cm–1. Exact mass: 328.11, MS (EI): m/z (%); 329 (45, M+1), 115 (100). Elemental analysis calculated (%) for; C14H20N2O5S·0.1H2O: C, 50.93; H, 6.17; N, 8.48; S, 9.71 Found: C, 50.77; H, 6.10; N, 8.439; S, 9.65.

##### 4.1.2.8. 4-Methyl-N-(2-(morpholinosulfonyl)ethyl)benzamide (8): CAS: 1216731-82-6.

A solution of 2-(morpholinosulfonyl)ethan-1-amine.HCl ( **D2** , 1.0 mmol) in water (10 mL) was treated with a solution of 4-methylbenzoic acid (1.0 mmol), CDI (1.0 mmol), and DIPEA (2.0 mmol) in dry ACN (8.0 mL) according to the general procedure B. The crude product was purified by silica gel column chromatography eluting with ethyl acetate:hexane (1:3) and crystallized from isopropanol:ethanol to afford the title compound 8 as a white solid (0.078 g, 25%); Rf = 0.12 (ethyl acetate:hexane 2:1); m.p. 174.2–175.2 °C; 1H NMR (400 MHz, DMSO-d6): δ: 2.39 (m, 3H, -CH3), 3.20–3.21 (m, 4H, Aliph-H), 3.33–3.35 (m, 2H, Aliph-H), 3.67–3.68 (m, 6H, Aliph-H), 7.31 (d, J = 7.9 Hz, 2H, Ar-H), 7.78 (d, J = 7.9 Hz, 2H, Ar-H), 8.60–8.62 (m, 1H, -CONH-). 13C NMR (100 MHz, DMSO-d6) δ: 21.8 (-CH3), 34.7 (Aliph-C), 46.1 (2 × Aliph-C), 47.7 (Aliph-C), 66.7 (2 × Aliph-C), 128.1 (2 × Ar-C), 129.8 (2 × Ar-C), 132.1 (Ar-C), 142.2 (Ar-C), 167.1 (C=O). FT-IR υmaks (cm-1): 3338 (N-H stretching), 1663 (amide I band), 1548 (amide II band) 1322 (SO2 asymmetric stretching), 1109 (SO2 symmetric stretching) cm–1. Exact mass: 312.11, MS (EI): m/z (%); 313 (10, M+1), 226 (100). Elemental analysis calculated (%) for C14H20N2O4S: C, 53.83; H, 6.45; N, 8.97; S, 10.26. Found: C, 53.75; H, 6.09; N, 8.83; S, 10.30.

##### 4.1.2.9. N-(2-sulfamoylethyl)benzamide (9): CAS: 4392-11-4

A solution of 2-aminoethane-1-sulfonamide.HCl ( **D3** , 1 mmol) in water (10 mL) was treated with a solution of benzoic acid (1.0 mmol), CDI (1.0 mmol), and DIPEA (2.0 mmol) in dry ACN (8.0 mL) according to the general procedure B. The crude product was purified by silica gel column chromatography eluting with ethyl acetate:hexane (3:1) to afford the title compound 9 as a white solid (0.079 g, 35%); Rf = 0,19 (ethyl acetate:hexane 3:1); m.p. 162–163 °C (previously reported as 165–166 °C [35]); 1H NMR (400 MHz, DMSO-d6): δ: 3.27–3.30 (m, 2H, Aliph-H), 3.67–3.72 (m, 2H, Aliph-H), 6.97 (s, 2H, -SO2NH2), 7.49–7.59 (m, 3H, Ar-H), 7.87 (d, 2H, J = 7.6 Hz, Ar-H), 8.62 (m, H,- -CONH-). 13C NMR (100 MHz, DMSO-d6) δ: 35.6 (Aliph-C), 54.5 (Aliph-C), 128.1 (2 × Ar-C), 129.2 (2 × Ar-C),132.2 (Ar-C), 135.1 (Ar-C), 167.3 (C=O). FT-IR υmaks (cm-1): 3382, 3331, 3266 (N-H stretching); 1635 (amide I band); 1529 (Amide II band); 1294 (SO2 asymmetric stretching); 1133 (SO2 symmetric stretching) cm–1. Exact mass: 228.06, MS (EI): m/z (%); 229 (10, M+1), 115 (100), 74 (18), 55 (5). Elemental analysis calculated (%) for C9H12N2O3S: C, 47.36; H, 5.30; N, 12.27; S, 14.05. Found: C, 47.07; H, 5.21; N, 12.34; S, 13.64.

##### 4.1.2.10. 4-Chloro-N-(2-sulfamoylethyl)benzamide (10): CAS: 1249977-51-2.

A solution of 2-aminoethane-1-sulfonamide.HCl ( **D3** , 1 mmol) in water (10 mL) was treated with a solution of 4-chlorobenzoic acid (1.0 mmol), CDI (1.0 mmol), and DIPEA (2.0 mmol) in dry ACN (8.0 mL) according to the general procedure B. The crude product was purified by silica gel column chromatography eluting with ethyl acetate:hexane:methanol (3:1:0.5) and triturated from ethyl acetate:hexane to afford the title compound 10 as a white solid (0.057 g, 22%); Rf = 0.63 (ethyl acetate:hexane:methanol 3:1:0.5); m.p. 158–159 °C; 1H NMR (400 MHz, DMSO-d6) δ: 3.20–3.24 (m, 2H, Aliph-H), 3.59–3.65 (m, 2H, Aliph-H), 6.90 (s, 2H, -SO2NH2), 7.53 (d, J = 8.4 Hz, 2H, Ar-H), 7.82 (d, J = 8.4 Hz, 2H, Ar-H), 8.64 (t, J = 4.3 Hz, 1H, -CONH-). 13C NMR (100 MHz, DMSO-d6) δ: 35.7 (Aliph-C), 54.4 (Aliph-C), 129.3 (2 × Ar-C), 129.9 (2 × Ar-C), 133.8 (Ar-C), 137.1 (Ar-C), 166.3 (C=O). FT-IR υmaks (cm–1): 3397, 3314, 3189 (N-H stretching); 1636 (amide I band); 1532 (Amide II band); 1278 (SO2 asymmetric stretching), 1143 (SO2 symmetric stretching) cm–1. Exact mass: 262.02, MS (EI): m/z (%); 263 (15, M+1), 115 (100), 74 (15), 55 (7). Elemental analysis calculated (%) for C9H11ClN2O3S·0.15C4H8O2: C, 41.79; H, 4.46; N, 10.15; S, 11.62. Found: C, 42.06; H, 4.26; N, 10.49; S, 11.40.

##### 4.1.2.11. 4-Methoxy-N-(2-sulfamoylethyl)benzamide (11): CAS: 1281521-12-7.

A solution of 2-aminoethane-1-sulfonamide.HCl ( **D3** , 1 mmol) in water (10 mL) was treated with a solution of 4-methoxybenzoic acid (1.0 mmol), CDI (1.0 mmol), and DIPEA (2.0 mmol) in dry ACN (8.0 mL) according to the general procedure B. The crude product was purified by silica gel column chromatography eluting with ethyl acetate:hexane:methanol (3:1:0.2) and crystallized from acetonitrile:water to afford the title compound 11 as a white solid (0.077 g, 30%); Rf = 0.5 (ethyl acetate:hexane 3:1:0.2); m.p. 176–177 °C; 1H NMR (400 MHz, DMSO-d6): δ: 3.25–3.29 (m, 2H, Aliph-H), 3.65–3.70 (m, 2H, Aliph-H), 3.79 (s, 3H, -OCH3), 6.95 (s, 2H, -SO2NH2), 7.03 (d, J = 8.5 Hz, 2H, Ar-H), 7.84 (d, J = 8.6 Hz, 2H, Ar-H), 8.47 (t, J = 5.4 Hz, 1H, -CONH-). 13C NMR (100 MHz, DMSO-d6) δ: 35.6 (Aliph-C), 54.7 (Aliph-C), 56.3 (-OCH3), 114.5 (2 × Ar-C), 127.3 (Ar-C), 129.9 (2 × Ar-C), 162.6 (Aliph-C), 166.8 (C=O). FT-IR υmaks (cm-1): 3378, 3264 (N-H stretching); 1613 (amide I band); 1551, 1509 (Amide II band); 1322 (SO2 asymmetric stretching); 1140 (SO2 symmetric stretching) cm–1. Exact mass: 258.07, MS (EI): m/z (%); 259 (100, M+1), 204 (9), 135 (47), 74 (12). Elemental analysis calculated (%) for C10H14N2O4S·0.2C2H3N: C, 46.87; H, 5.52; N, 11.56; S, 12.03. Found: C, 47.00; H, 5.13; N, 11.17; S, 11.65.

##### 4.1.2.12. 4-Methyl-N-(2-sulfamoylethyl)benzamide (12): CAS: 1249082-36-7.

A solution of 2-aminoethane-1-sulfonamide.HCl ( **D3** , 1 mmol) in water (10 mL) was treated with a solution of 4-methylbenzoic acid (1.0 mmol), CDI (1.0 mmol), and DIPEA (2.0 mmol) in dry ACN (8.0 mL) according to the general procedure B. The crude product was purified by silica gel column chromatography eluting with ethyl acetate:hexane:methanol (3:1:0.5) and crystallized from ethyl acetate:hexane to afford the title compound 12 as a white solid (0.073 g, 30%); Rf = 0.68 (ethyl acetate:hexane:methanol 3:1:0.5); m.p. 181–182 °C; 1H NMR (400 MHz, DMSO-d6) δ: 2.39 (3H, s, -CH3) 3.25–3.29 (m, 2H, Aliph-H), 3.66–3.70 (m, 2H, Aliph-H), 6.96 (s, 2H, -SO2NH2), 7.31 (d, J = 7.6 Hz, 2H, Ar-H), 7.77 (d, J = 7.0 Hz, 2H, Ar-H), 8.55 (m, 1H, -CONH-). 13C NMR (100 MHz, DMSO-d6) δ: 21.9 (-CH3), 35.6 (Aliph-C), 54.6 (Aliph-C), 128.1 (2 × Ar-C), 129.8 (2 × Ar-C), 132.3 (Ar-C), 142.1 (Ar-C), 167.2 (C=O). FT-IR υmaks (cm–1): 3373, 3323, 3266 (N-H stretching); 1634 (amide I band); 1533 (Amide II band); 1297 (SO2 asymmetric stretching); 1133 (SO2 symmetric stretching) cm–1. Exact mass: 242.07, MS (EI): m/z (%); 243 (5, M+1), 225 (62), 115 (100), 74 (13), 55 (7). Elemental analysis calculated (%) for C10H14N2O3S·0.05C4H8O2: C, 49.66; H, 5.88; N, 11.36; S, 13.00. Found: C, 49.53; H, 5.66; N, 11.53; S, 12.68.

##### 4.1.2.13. N-(2-(N-(4-methoxyphenyl)sulfamoyl)ethyl)benzamide (13): CAS: 1327651-78-4

A solution of 2-amino- *N* -(4-methoxyphenyl)ethane-1-sulfonamide.HCl ( **D4** , 1 mmol) in water (10 mL) was treated with a solution of benzoic acid (1.0 mmol), CDI (1.0 mmol), and DIPEA (2.0 mmol) in dry ACN (8.0 mL) according to the general procedure B. The crude product was purified by silica gel column chromatography eluting with ethyl acetate:hexane (1:1) and crystallized from ethyl acetate:diethyl ether to afford the title compound 13 as a white solid (0.076 g, 23%); Rf = 0.26 (ethyl acetate:hexane 1:1); m.p. 110–111 °C; 1H NMR (400 MHz, DMSO-d6) δ: 3.27–3.31 (m, 2H, Aliph-H), 3.65–3.70 (m, 2H, Aliph-H), 3.76 (s, 3H,-OCH3), 6.93 (d, J = 9 Hz, 2H, Ar-H), 7.22 (d, J = 9 Hz, 2H, Ar-H), 7.48–7.51 (m, 1H, Ar-H), 7.55–7.58 (m, 2H, Ar-H), 7.82–7.84 (m, 2H, Ar-H), 8.59–8.62 (m, 1H, -CONH-), 9.56 (brs, 1H, -SO2NH-). 13C NMR (100 MHz, DMSO-d6) δ: 34.7 (Aliph-C), 49.5 (Aliph-C), 55.7 (-OCH3), 114.9 (3 × Ar-C), 123.9 (2 × Ar-C), 127.6 (2 × Ar-C), 130.8 (2 × Ar-C), 131.8 (Ar-C), 134.4 (Ar-C), 157.1 (Ar-C), 166.8 (C=O). FT-IR υmaks (cm–1): 3325 (N-H stretching), 1625 (amide I band), 1537 (amide II band), 1313 (SO2 asymmetric stretching), 1141 (SO2 symmetric stretching) cm–1. Exact mass: 334.10, MS (EI): m/z (%); 335 (100, M+1), 271 (33), 266 (22), 253 (12), 225 (16), 212 (23), 184 (6), 148 (12), 115 (30), 100 (67). Elemental analysis calculated (%) for C16H18N2O4S: C, 57.47; H, 5.43; N, 8.38; S, 9.59. Found: C, 57.67; H, 5.20; N, 8.42; S, 9.52.

##### 4.1.2.14. 4-Chloro-N-(2-(N-(4-methoxyphenyl)sulfamoyl)ethyl)benzamide (14):

A solution of 2-amino- *N* -(4-methoxyphenyl)ethane-1-sulfonamide.HCl ( **D4** , 1 mmol) in water (10 mL) was treated with a solution of 4-chlorobenzoic acid (1.0 mmol), CDI (1.0 mmol), and DIPEA (2.0 mmol) in dry ACN (8.0 mL) according to the general procedure B. The crude product was purified by silica gel column chromatography eluting with ethyl acetate:hexane (1:1) and crystallized from isopropanol:water to afford the title compound 14 as a white solid (0.103 g, 28%); Rf = 0.29 (ethyl acetate:hexane 1:1); m.p. 160–161 °C; 1H NMR (400 MHz, DMSO-d6) δ: 3.27–3.30 (m, 2H, Aliph-H), 3.64–3.69 (m, 2H, Aliph-H), 3.76 (s, 3H, -OCH3), 6.93 (d, J = 8.9 Hz, 2H, Ar-H), 7.21 (d, J = 8.9 Hz, 2H, Ar-H), 7.57 (d, J = 8.6 Hz, 2H, Ar-H), 7.84 (d, J = 8.6 Hz, 2H, Ar-H), 8.68–8.71 (m, 1H, -CONH-), 9.56 (brs, 1H, -SO2NH-). 13C NMR (100 MHz, DMSO-d6) δ: 34.7 (Aliph-C), 49.6 (Aliph-C), 55.7 (-OCH3), 114.9 (2 × Ar-C), 123.9 (2 × Ar-C), 128.9 (2 × Ar-C), 129.5 (2 × Ar-C), 130.8 (Ar-C), 133.2 (Ar-C), 136.7 (Ar-C), 157.1 (Ar-C), 165.7 (C=O). FT-IR υmaks (cm–1): 3310, 3272 (N-H stretching), 1643 (amide I band), 1547 (amide II band), 1309 (SO2 asymmetric stretching), 1144 (SO2 symmetric stretching) cm–1. Exact mass: 368.06, MS (EI): m/z (%); 369 (63, M+1), 305 (10), 287 (6), 165 (5), 143 (29), 115 (100), 100 (50), 74 (8), 55 (20). Elemental analysis calculated (%) for C16H17ClN2O4S·0.15H2O: C, 51.72; H, 4.69; N, 7.54; S, 8.63. Found: C, 51.63; H, 4.50; N, 7.62; S, 8.53.

##### 4.1.2.15. 4-Methoxy-N-(2-(N-(4-methoxyphenyl)sulfamoyl)ethyl)benzamide (15):

A solution of 2-amino- *N* -(4-methoxyphenyl)ethane-1-sulfonamide.HCl ( **D4** , 1 mmol) in water (10 mL) was treated with a solution of 4-methoxybenzoic acid (1.0 mmol), CDI (1.0 mmol), and DIPEA (2.0 mmol) in dry ACN (8.0 mL) according to the general procedure B. The crude product was purified by silica gel column chromatography eluting with ethyl acetate:hexane (3:1) and crystallized from aceton:diethyl ether to afford the title compound 15 as a white solid (0.105 g, 29%); Rf = 0.5 (ethyl acetate:hexane 3:1); m.p. 112–113°C; 1H NMR (400 MHz, DMSO-d6) δ: 3.25–3.28 (m, 2H, Aliph-H), 3.62–3.67 (m, 2H, Aliph-H), 3.76 (s, 3H, -OCH3), 3.84 (s, 3H, -OCH3), 6.92–6.94 (m, 2H, Ar-H), 7.01–7.03 (m, 2H, Ar-H), 7.20–7.22 (m, 2H, Ar-H), 7.80 (q, J = 2.0/5.0 Hz, 2H, Ar-H), 8.46 (t, J = 5.5 Hz, 1H, -CONH-), 9.54 (brs, 1H, -SO2NH-). 13C NMR (100 MHz, DMSO-d6) δ: 35.0 (Aliph-C), 50.3 (Aliph-C), 56.1 (-OCH3), 56.2 (-OCH3), 114.4 (2 × Ar-C), 115.4 (2 × Ar-C), 124.4 (2 × Ar-C), 127.1 (2 × Ar-C), 129.9 (Ar-C), 131.2 (Ar-C), 157.5 (Ar-C), 162.6 (Ar-C), 166.7 (C=O). FT-IR υmaks (cm–1): 3346, 3206 (N-H stretching), 1630 (amide I band), 1540 (amide II band), 1301 (SO2 asymmetric stretching), 1128 (SO2 symmetric stretching) cm–1. Exact mass: 364.11, MS (EI): m/z (%); 365 (100, M+1), 301 (10), 242 (6), 178 (3.5), 135 (11), 115 (90), 83 (77), 55 (13), 41 (5). Elemental analysis calculated (%) for C17H20N2O5S·0.15C3H6O: C, 56.17; H, 5.65; N, 7.51; S, 8.59. Found: C, 56.49; H, 5.59; N, 7.80; S, 8.37.

##### 4.1.2.16. N-(2-(N-(4-methoxyphenyl)sulfamoyl)ethyl)-4-methylbenzamide (16):

A solution of 2-amino- *N* -(4-methoxyphenyl)ethane-1-sulfonamide.HCl ( **D4** , 1 mmol) in water (10 mL) was treated with a solution of 4-methylbenzoic acid (1.0 mmol), CDI (1.0 mmol), and DIPEA (2.0 mmol) in dry ACN (8.0 mL) according to the general procedure B. The crude product was purified by silica gel column chromatography eluting with ethyl acetate:hexane (2:1) and crystallized from ethyl acetate:diethyl ether to afford the title compound 16 as a white solid (0.202 g, 58%); Rf = 0.26 (ethyl acetate:hexane 2:1); m.p. 127–128 °C; 1H NMR (400 MHz, DMSO-d6) δ: 2.38 (s, 3H, -CH3), 3.25–3.29 (m, 2H, Aliph-H), 3.63–3.68 (m, 2H, Aliph-H), 3.76 (s, 3H, -OCH3), 6.93 (d, J = 8.5 Hz, 2H, Ar-H), 7.21 (d, J = 8.5 Hz, 2H, Ar-H), 7.29 (d, J = 8.0 Hz, 2H, Ar-H), 7.73 (d, J = 7.9 Hz, 2H, Ar-H), 8.51–8.54 (m, 1H, -CONH-), 9.50 (s, 1H, -SO2NH-). 13C NMR (100 MHz, DMSO-d6) δ: 21.8 (Aliph-C), 35.1 (Aliph-C), 50.2 (Aliph-C), 56.1 (-OCH3), 115.4 (2 × Ar-C), 124.4 (2 × Ar-C), 128.1 (2 × Ar-C), 129.7 (2 × Ar-C), 131.3 (Ar-C), 132.1 (Ar-C), 142.2 (Ar-C), 157.6 ( Ar-C), 167.1 (C=O). FT-IR υmaks (cm–1): 3377, 3325 (N-H stretching), 1625 (amide I band), 1540 (amide II band), 1314 (SO2 asymmetric stretching), 1143 (SO2 symmetric stretching) cm–1. Exact mass: 348.11, MS (EI): m/z (%); 349 (100, M+1), 285 (10), 226 (16), 162 (10), 115 (90), 83 (86), 55 (15), 41 (5). Elemental analysis calculated (%) for C17H20N2O4S: C, 58.60; H, 5.79; N, 8.04; S, 9.20. Found: C, 58.34; H, 5.49; N, 8.04; S, 8.92.

### 4.2. Biological methods

#### 4.2.1. Cell culture and treatments

The human neuroblastoma cells (SH-SY5Y), triple-negative breast cancer cells (MDA-MB-231), and human pancreatic cancer cells (Panc1) used in this study were purchased from the American Type Culture Collection (ATCC). The cells were suspended in complete Dulbecco’s modified Eagle medium (DMEM) (Life Technologies, Gibco BRL, Grand Island, NY) supplemented with 10% fetal bovine serum (FBS, Hyclone), 1% penicillin and streptomycin (100 U/mL, Invitrogen) and plated in cell culture dishes. The cultures were maintained at 37 °C in a 5% CO2 95% humidified atmosphere. After reaching 85% confluence, the cells were transferred to 96-well plates or culture dishes. For MTT assays, the cells were seeded into 96-well cell culture plates at a density of 2 × 103 cells per well and allowed to adhere for 24 h. The cells were treated with fresh medium containing experimental compounds (100, 10, 1 µM) and incubated in a 5% CO2 incubator for 48 and 72 h. For protein analysis, the cells were seeded into 6-well cell culture plates at a density of 0.5 x 106 cells/well and allowed to adhere to the surface for 24 h. The stock solutions of the test compounds (5 mM) were prepared in sterile DMSO, and these stocks were then appropriately diluted with the complete culture medium. DMSO levels were maintained below 1% in the test concentrations.

#### 4.2.2. MTT assay

Briefly, following exposures, MTT (at a final concentration of 0.5 mg/mL) was added to each well, and plates were incubated for 2 h at 37 °C in a 5% CO2 humidified incubator. The reaction mixture was removed, and DMSO was added to each well. The plates were shaken at room temperature, and the absorbance was measured at 570 nm and 630 nm using a microplate reader (VersaMax, Molecular Devices, USA). Percent survival was plotted relative to vehicle control cells, which were normalized to 100% survival.

#### 4.2.3. Protein analysis

The cells (0.5 × 106 cell/well) were seeded into 6 well plates and incubated for 24 h, and then treated with selected experimental compounds for 24 h and 48 h. Following incubation, the cells were washed with ice-cold 1X PBS and lysed in 1X cell lysis buffer. These lysates were quantified using the BCA protein assay. Then, the cell lysates were analyzed by western blot.

#### 4.2.4. Western blotting

On a 12% (w/v) tris-glycine denaturing gel, 20 μg protein was separated by electrophoresis, and then transferred to a PVDF membrane. After blocking, the membrane was incubated with primary antibodies, anti-Bax rabbit monoclonal antibody, anti-Bcl-2 rabbit monoclonal antibody, anti-Bcl-xL rabbit monoclonal antibody, anti-caspase-3 rabbit monoclonal antibody, and anti-caspase-9 rabbit monoclonal antibody (1:1000, Cell Signaling Technology) at 4 °C overnight. Following the incubation, the membrane was incubated with an anti-β-actin mouse monoclonal antibody (1:1000, Cell Signaling Technology) at room temperature for 1 h. After washing, the membrane was incubated with peroxidase-conjugated secondary antibodies for 1 h to visualize labeled proteins by enhanced chemiluminescence.

#### 4.2.5. Annexin V FITC/PI test

Th e cells were treated with cisplatin or compound 7, 14, or 15 at their respective cytotoxic doses against three cancer cell lines. The cells were harvested after 48 h, washed with PBS, and analyzed using the Annexin V FITC/PI Apoptosis Detection kit (BioVision, Mountain View, CA, USA) with a benchtop flow cytometer (Accuri C6 flow cytometry, Becton Dickinson). While the lower right quadrant shows the early apoptotic cells (FITC positive and PI negative) indicating Annexin V binding and cytoplasmic membrane integrity, the upper right quadrant shows the late apoptotic cells (positive for FITC and PI). The upper left quadrant displays the necrotic cells, negative for FITC, and showing PI uptake.

#### 4.2.6. Statistical analysis

Data were expressed as means ± standard deviation (SD) for five (MTT) or three (western blotting) independent experiments. Comparisons of means between groups were performed by one-way analysis of variance (ANOVA) followed by Tukey’s post hoc test. Statistical significance was assigned at P < 0.05.

## References

[ref1] (2018). GLOBOCAN estimates of incidence and mortality worldwide for 36 cancers in 185 countries. Global cancer statistics.

[ref2] (2017). The different mechanisms of cancer drug resistance: A brief review. Advanced Pharmaceutical Bulletin.

[ref3] (2009). Anticancer drug development unique aspects of pharmaceutical development. Pharmaceutical Perspectives of Cancer Therapeutics.

[ref4] (2011). Hallmarks of cancer: The next generation. Cell.

[ref5] (2016). Major apoptotic mechanisms and genes involved in apoptosis. Tumor Biology.

[ref6] (2016). A fate worse than death: Apoptosis as an oncogenic process. Nature Reviews Cancer.

[ref7] (2014). -1197: A novel and specific Bcl-2/Bcl-xL inhibitor inducing complete and long-lasting tumor regression in vivo. PLoS ONE.

[ref8] (2016). Derivatives of procaspase-activating compound 1 (PAC-1) and their anticancer activities. Current Medicinal Chemistry.

[ref9] (2009). Taurine protects transformed rat retinal ganglion cells from hypoxia-induced apoptosis by preventing mitochondrial dysfunction. Brain Research.

[ref10] (2015). A review on the biomedical importance of taurine. International Journal of Pharma Research and Health Sciences.

[ref11] (2014). Mechanism of taurine-induced apoptosis in human colon cancer cells. Acta Biochimica et Biophysica Sinica.

[ref12] (2018). Effect of taurine on cell proliferation and apoptosis human lung cancer A549 cells. Oncology Letters.

[ref13] (2009). Taurine - a possible fingerprint biomarker in non-muscle invasive bladder cancer: a pilot study by 1H NMR spectroscopy. Cancer Biomarkers.

[ref14] (2011). Abd El Hameed OM. Angiogenesis.

[ref15] (1983). Novel anticonvulsant taurine derivatives. Progress in Clinical and Biological Research.

[ref16] (2010). The evolving role of taurolidine in cancer therapy. Annals of Surgical Oncology.

[ref17] (1989). Pharmacokinetics of tauromustine in cancer patients. Cancer Chemotherapy and Pharmacology.

[ref18] (2001). Taurolidine: Cytotoxic and mechanistic evaluation of a novel antineoplastic agent. Cancer Research.

[ref19] (1947). Derivatives of taurine and β-Alanine 1. Journal of the American Chemical Society.

[ref20] (2006). Taurine analogues and taurine transport: therapeutic advantages. Taurine 6.

[ref21] (2002). The use of taurine analogues to investigate taurine functions and their potential therapeutic applications. Amino acids.

[ref22] (1947). Studies in Chemotherapy. XV. Amides of Pantoyltaurine 1. Journal of the American Chemical Society.

[ref23] (2010). Synthesis and QSAR of quinazoline sulfonamides as highly potent human histamine H 4 receptor inverse agonists. Journal of Medicinal Chemistry.

[ref24] (2003). Intermediates in the Ing-Manske reaction. Arkivoc.

[ref25] (2017). and antimicrobial activity of some taurinamide derivatives. Marmara Pharmaceutical Journal.

[ref26] (2007). Synthesis and anticonvulsant activity of some N-Phenyl-2-phtalimidoethanesulfonamide derivatives. Archiv der Pharmazie.

[ref27] (2018). Novel benzotriazole N-acylarylhydrazone hybrids: Design, synthesis, anticancer activity, effects on cell cycle profile, caspase-3 mediated apoptosis and FAK inhibition. Bioorganic Chemistry.

[ref28] (2016). Apoptosis as anticancer mechanism: Function and dysfunction of its modulators and targeted therapeutic strategies. Aging.

[ref29] (2017). From basic apoptosis discoveries to advanced selective BCL-2 family inhibitors. Nature Reviews Drug Discovery.

[ref30] (2017). Selenopheno quinolinones and coumarins promote cancer cell apoptosis by ROS depletion and caspase-7 activation. Life Sciences.

[ref31] (2017). A comparative study of amide-bond forming reagents in aqueous media – Substrate scope and reagent compatibility. Tetrahedron Letters.

[ref32] (2020). Unconventional amino acids in medicinal chemistry: first report on taurine merged within carbonic anhydrase inhibitors. Bioorganic Chemistry.

[ref33] (1983). Anticonvulsant activity of some 2-aminoethanesulphonic acid (taurine) derivatives. European Journal of Pharmacology.

[ref34] (2009). an inexpensive, convenient, and practical 13C NMR solvent for strong polar amino acids and their derivatives. Letters in Organic Chemistry.

[ref35] (1940). The preparation of some amino sulfonamides. Journal of the American Chemical Society.

[ref36] (1984). Synthesis and anticonvulsant properties of some 2-aminoethanesulfonic acid (taurine) derivatives. Journal of Pharmaceutical Sciences.

